# Supplementary material for: Deletion of wfs1 Impairs Oligodendrocyte Precursor Cells Dorsal Distribution and Myelination Through the wfs1-hmgcs1 Axis in Zebrafish
Source: Biology (Basel). 2026 Aug 16;15(16):1402. doi: 10.3390/biology15161402 (PMC13510151; doi:10.3390/biology15161402)
Supplement: Supplementary file 1 [file biology-15-01402-s001.zip › File S1-Transcriptome sequencing data and differentially expressed genes (DEGs) in wfs1-- zebrafish at 5 dpf/2.KEGG/wfs1KOvsWT/UP/src/dre04914.html]

dre04914


- dre:245702

- Up regulated genes

245702(1.27755220735994)

- dre:568726

- Up regulated genes

568726(1.14419230813088)
- dre:560807

- Up regulated genes

560807(1.71247301071773)

- dre:100000252

- Up regulated genes

100000252\_3(1.94434154854557) 100000252(1.09163460881879)

- dre:280649

- Up regulated genes

280649\_1(2.27023747569103)

- dre:767751

- Up regulated genes

767751\_1(3.05541031760882)

- dre:100009657

- Up regulated genes

100009657\_1(1.89754653566672)

- dre:323509

- Up regulated genes

323509\_1(2.61236898874666)

- dre:562552

- Up regulated genes

562552(1.50682290794657)

- dre:100000252

- Up regulated genes

100000252\_3(1.94434154854557) 100000252(1.09163460881879)

Close
